# Prevalence and genotype distribution of human papillomavirus infection among women undergoing HPV testing at a hospital in Zhangzhou, China: a retrospective study

**DOI:** 10.3389/fpubh.2026.1907680

**Published:** 2026-09-23

**Authors:** Wenxin Xu, Yueli Guo, Weide Chen, Guowei Wang, Qingyu Wu, Yuanjun Zeng

**Affiliations:** 1 Collaborative Innovation Center for Translation Medical Testing and Application Technology, Department of Medical Technology, Zhangzhou Health Vocational College, Zhangzhou, Fujian, China; 2 Department of Laboratory Medicine, Zhangzhou Affiliated Hospital of Fujian Medical University, Zhangzhou, Fujian, China; 3 State Key Laboratory of Vaccines for Infectious Diseases, Xiang An Biomedicine Laboratory, Xiamen University, Xiamen, Fujian, China

**Keywords:** age distribution, China, genotype, human papillomavirus, prevalence

## Abstract

**Introduction:**

This retrospective, single-center study aims to analyze the prevalence and genotype distribution of human papillomavirus (HPV) infection in women undergoing HPV testing at a hospital in Zhangzhou, China.

**Methods:**

A total of 13,492 females, 18–85 years, undergoing HPV testing at the physical examination center of a hospital in Zhangzhou, China, were recruited. Cervical cast-off cells were collected for HPV testing by low-density gene chip and flow hybridization technology using HPV typing test kit. A total of 27 kinds of HPV types were detected using this method, including 17 kinds of high-risk HPV (HR-HPV) and 10 kinds of low-risk/uncertain oncogenic significance HPV (LR-HPV).

**Results:**

The overall HPV infection rate was 16.86% (2,275/13,492), with 12.59% (1,699/13,492) females detected as single HPV-type infection and 4.27% (576/13492) females detected as multiple HPV-type infection. Among all 27 detected HPV types, HPV52 exhibited the highest prevalence (3.58%, 483/13,492), followed by HPV53 (1.76%, 237/13,492) and HPV81 (1.71%, 231/13,492). The most common HPV coinfection pattern was HPV52 + HPV53 (12/2,275). Overall HPV prevalence showed a “two-peak” age-related distribution, with the first peak in the ≤29-year age group (19.69%) and the second peak in the ≥60-year age group (24.45%), respectively. It decreased in the 40- to 49-year age group (13.64%). The prevalence of single/multiple HPV and HR-HPV/LR-HPV infection also exhibited age-related trends similar to those described above. Binary logistic regression analysis revealed a non-linear relationship between HPV prevalence and age (*p* < 0.05).

**Conclusion:**

Our study revealed the basic genotype- and age-specific distribution of HPV infection among females attending the physical examination center of a hospital in Zhangzhou. These results emphasized that the specific population should strengthen regular HPV surveillance and improve awareness of vaccination, especially for the highly prevalent HPV types (HPV52,53,51). In addition, our findings provide valuable insights for the measurement of HPV prevention interventions.

## 1 Introduction

Cervical cancer ranks as the fourth most prevalent cancer among females across the world, contributing an estimated 660,000 newly diagnosed cases and 350,000 disease-related mortalities globally in 2022 (1). Cervical cancer ranked sixth in mortality among all female malignancies in China in 2022, with an estimated 150,700 new cervical cancer cases and 55,700 related deaths (2). Persistent infection with high-risk human papillomavirus (HR-HPV) has been identified as a risk factor for cervical intraepithelial neoplasia (CIN) and cervical cancer. The World Health Organization proposed a 90–70–90 global strategy to accelerate the elimination of cervical cancer by 2030, which is to achieve HPV vaccination rates of ≥90% in women before the age of 15, efficient screening coverage rates of ≥70% in women before the age of 35 and 45, and treatment rates of ≥90% for diagnosed cervical lesions (3).

Over 200 types of HPV have been identified. According to the International Agency for Research on Cancer (IARC) Working Group HPV classification framework, 12 HPV types, including HPV16, 18, 31, 33, 35, 39, 45, 51, 52, 56, 58, and 59, have been classified as carcinogenic (IARC Group 1) to humans, other eight HPV types in this high-risk clade, including 26, 53, 66, 67, 68, 70, 73, and 82, were classified as probable/possible carcinogenic (IARC Group 2A and IARC Group 2B). In addition, HPV types 6, 11, 40, 42, 43, 44, 54, 61, 72, 81, and 89 were classified as non-oncogenic (IARC Group 3). Currently, the HPV vaccine is still the most effective strategy to prevent HPV infection and cervical cancer. In addition, routine HPV screening, especially HR-HPV screening, is strongly recommended, which can contribute to the prevention of cervical cancer (4, 5).

Previously, a nationwide study in China revealed that the overall HPV prevalence of Chinese females was at a relatively high level (17.70%), with high- and low-risk types being 13.12 and 4.58%, respectively (6). Several studies have demonstrated that HPV prevalence among women and the distribution of predominant HPV genotypes exhibit distinct geographical disparities and genotypic diversity. A study in Fujian Province demonstrated that HR-HPV infections were mainly attributed to HPV16, HPV52, HPV58, and HPV53 types (7). Zhangzhou City is located in southeastern China, Fujian Province. However, the prevalence and infection patterns of HPV among females in Zhangzhou City have not been reported yet. Therefore, it is necessary to obtain the local baseline data of HPV prevalence in Zhangzhou City, which will contribute to the prevention of HPV infection and the implementation of the HPV vaccination program.

In this study, we aim to investigate the local genotype-specific prevalence of HPV infection and analyze the age-related trends of HPV infection among females undergoing HPV testing in a physical examination center of a hospital in Zhangzhou City.

## 2 Materials and methods

### 2.1 Ethics statement

The study involving humans was approved by the Ethics Committee of Zhangzhou Affiliated Hospital of Fujian Medical University (Ethics approval number: 2026LWB202) on 4 June 2026. The study was conducted in accordance with the local legislation and institutional requirements. Written informed consent for participants was not required from the participants or the participants’ legal guardians/next of kin because this is a retrospective study, and all the participants’ results were retrospectively collected.

### 2.2 Study design and populations

Initially, a total of 23,650 participants with HPV testing data were retrospectively retrieved from the laboratory information system (LIS) at Zhangzhou Affiliated Hospital of Fujian Medical University, from January 2025 to August 2025. Participants who underwent HPV testing at the health examination center of a hospital in Zhangzhou were included (n = 13,492). Meanwhile, the participants attending other clinics in this hospital, including gynecology, urology, and obstetrics, were excluded (*n* = 10,029). We excluded male participants (*n* = 129). Duplicate records were checked using unique participant IDs. Among the 13,492 included participants, no duplicate subjects were observed. In addition, no cases with incomplete data were observed. Therefore, the number of final included participants was 13,492, aged 18–85 years. The participant selection flow diagram is shown in Figure 1.

**Figure 1:**
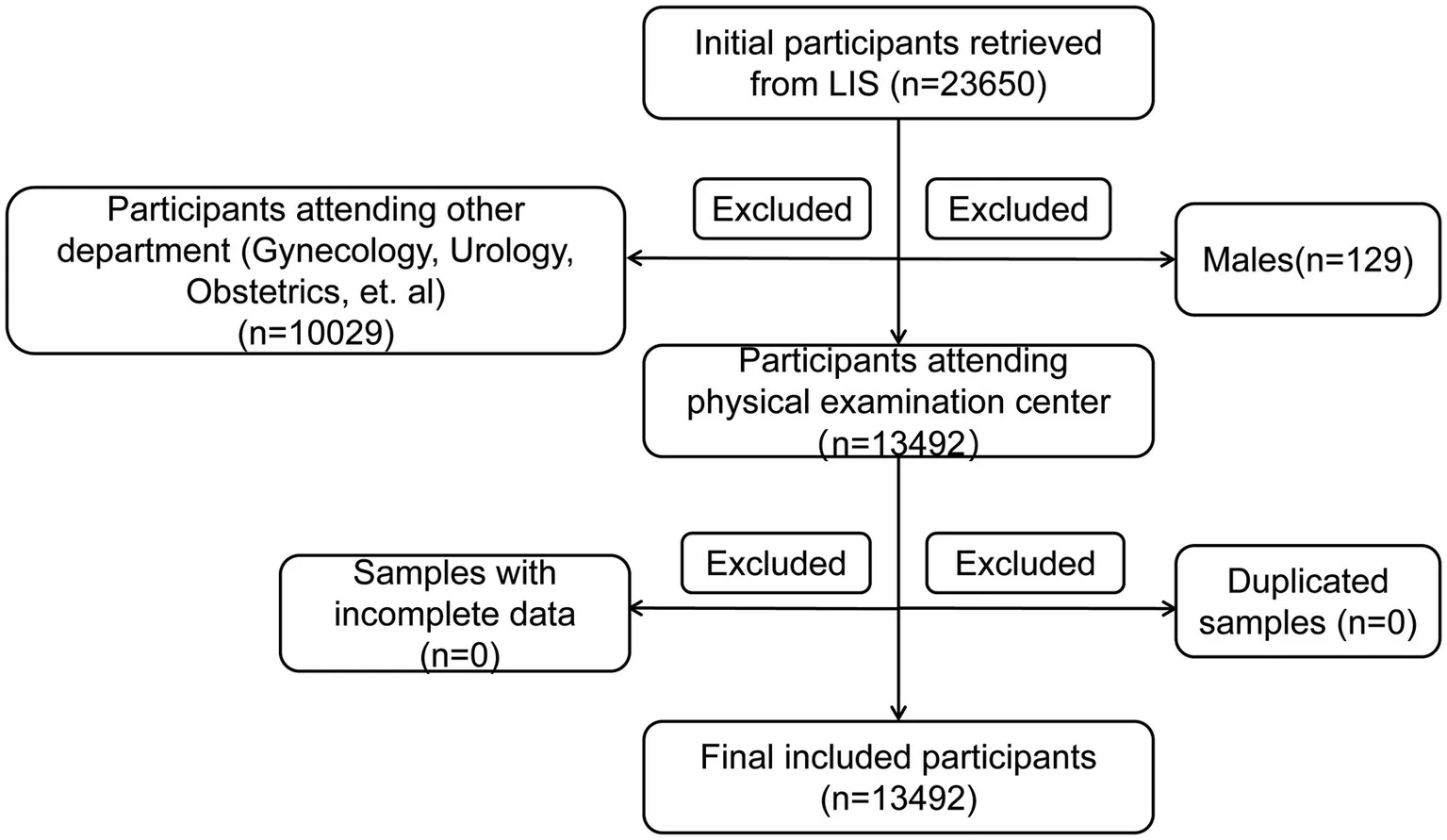
Participants’ selection flow diagram.

### 2.3 HPV genotyping

To collect the cervical cast-off cells, a vaginal dilator was used to expose the cervix. Cervical mucus was removed with a cotton swab. The cervical brush was placed at the cervical opening and gently rotated clockwise for 3–5 times. The cervical brush was slowly removed and placed into a sample tube containing cell preservation solution. The brush head was left in the sample tube, and the bottle cap was tightened. The sample tubes were labeled with number and date and then transported to the laboratory for immediate DNA extraction or stored at 2–8 °C for no more than 2 weeks.

To extract the HPV DNA, 0.5–1.0 mL of cell preservation solution containing cervical cast-off cells was centrifuged at 14,000 rpm for 1 min, and the supernatant was discarded. Then HPV DNA was extracted using cell lysis buffer (Kaipu Biotechnology, China, Catalog: 20150019) according to the manufacturer’s instructions. Then, 1 μL of extracted DNA sample was served as a template for polymerase chain reaction (PCR) amplification and flow-through hybridization technology using the HPV typing test kit (PCR + Membrane Hybridization Method) (Chaozhou Kaipu Biotechnology Co. Ltd., Catalog: 20143402188) and nucleic acid molecule rapid hybridization instrument (HHM-2, Chaozhou Kaipu Biotechnology Co. Ltd.) according to the manufacturer’s guidelines.

This testing method used PCR to amplify HPV DNA. The PCR products were then applied to a low-density gene chip, and flow-hybridization technology was used to detect hybridization between the amplified products and type-specific probe membranes; thereby, a total of 27 different HPV genotypes were detected.

According to the HPV classification framework of IARC, HPV types were classified into group 1 (carcinogenic to humans), group 2A (probably carcinogenic to humans), group 2B (possibly carcinogenic to humans), and group 3 (non-oncogenic HPV or uncertain oncogenic significance). In this study, the HPV types of IARC Group 1/2A/2B were classified as HR-HPV. Meanwhile, the HPV types of IARC Group 3 were classified as low-risk/uncertain oncogenic significance HPV (LR-HPV). Here, we detected 17 HR-HPV types (HPV16, HPV18, HPV26, HPV31, HPV33, HPV35, HPV39, HPV45, HPV51, HPV52, HPV53, HPV56, HPV58, HPV59, HPV66, HPV68, HPV82) and 10 LR-HPV types (HPV6, HPV11, HPV40, HPV42, HPV43, HPV44, HPV55, HPV61, HPV81, HPV83) using HPV kit.

### 2.4 Quality control and judgment criteria

(1) Limit of detection (LOD): The kit’s LOD is no higher than 5,000 copies per reaction.
(2) Specificity: The detection results of HPV negative standard samples should all be negative. In addition, the detection results of HPV-positive standard samples or HR-HPV samples should not exhibit any cross-reactions. The cross-reactivity rate in the detection of LR-HPV samples should be less than 10%.
(3) Quality control: Plasmid clones containing HPV DNA were used as the positive control samples. DNase-free H_2_O was used as the negative control sample. Each membrane has a biotin control point to detect the reaction between alkaline phosphatase and its substrate. In addition, the membrane contains an internal control point, which serves as an inner quality control template for DNA. If the internal control point is positive, the results are reliable.
(4) Judgment criteria: This reagent is a qualitative detection reagent. The positive test result is a clear and visible blue-purple dot. Based on the arrangement of various HPV probes on the membrane strip, the HPV type can be determined by the positive point. If the internal control point and biotin point are positive while the other points are negative, the test result is judged negative. If one or more HPV genotyping points are positive, the sample is judged as single- or multiple HPV positive.

Single genotype infection was defined as the infection of one HPV genotype among the 27 HPV types mentioned above; multiple infection is defined as the infection of ≥2 positive HPV types among the 27 HPV types mentioned above.

### 2.5 Statistical analysis

Statistical analysis was performed using SPSS software 26.0 (IBM Corp., NY, USA). The prevalence of age-specific or HPV type-specific infection was reported with Wilson score 95% confidence intervals (CI). Odds ratios (ORs) were estimated by comparing the HPV prevalence across different age groups with ≥60-year age group as reference; 95% confidence intervals for ORs were calculated by Woolf’s logit method. Chi-square or Fisher’s exact test was applied for the comparison of prevalence in different age groups with ≥60-year age group. For comparison between multiple age groups, *p*-value was adjusted using Bonferroni method; *p* < 0.05/10 = 0.005 was considered statistically significant.

Logistic regression analysis was performed with age and age squared (age^2^) as continuous variables to evaluate non-linear (quadratic) associations between the prevalence of HPV infection and age. HPV infection status (positive or negative) was included as the dependent variable, whereas age and age squared were included as independent variables. We included HPV infection status, age, and its quadratic term (age^2^) in the binary logistic regression model. Statistical significance of the quadratic term was set at a *p*-value of < 0.05.

GraphPad Prism 10 (Boston, MA, USA) was used for data visualization, and frequencies were used to calculate HPV types. The predicted logistic plot of logistic regression was produced by GraphPad Prism 10; a *p*-value of < 0.05 was considered statistically significant.

## 3 Results

### 3.1 Population characteristics and overall prevalence of HPV infection

The mean age of the total 13,492 included females was 47.27 ± 10.26 years. All females were divided into five age groups: (1) ≤ 29 years: 523 cases; (2) 30–39 years: 2,939 cases; (3) 40–49 years: 3,827 cases; (4) 50–59 years: 4,690 cases; and (5) ≥ 60 years: 1,513 cases.

A total of 2,275 females tested positive for HPV infection, corresponding to a positive rate of 16.86% (95% CI: 16.24–17.50%); 1,699 females (12.59, 95% CI: 12.03–13.15%) were infected with a single HPV genotype, whereas 576 females (4.27, 95% CI: 3.94–4.62%) were infected with multiple HPV genotypes.

Among 2,275 HPV-positive females, 18.24% (415/2275) females had co-infection with 2 HPV types, 5.27% females (120/2,275) had co-infection with 3 HPV types, 1.27% (29/2,275) were positive for 4 HPV types, 0.31% (7/2,275) were positive for 5 HPV types, 0.09% (2/2,275) were positive for 6 HPV types, 0.09% (2/2,275) were positive for 7 HPV types, and 0.044% (1/2,275) were positive for 9 HPV types (Table 1). In addition, the predominant HPV co-infection pattern was HPV52 + HPV53 (12 cases), followed by HPV52 + HPV58 (11 cases) and HPV42 + HPV52 (9 cases) (Table 2).

**Table 1:** Prevalence of multiple HPV coinfection patterns among females attending a hospital in Zhangzhou.

Number of HPV types in the HPV coinfection cases	*N* ^a^	*n* ^b^	%	95% CI
1	2,275	1,699	74.68	72.85–76.43
2	2,275	415	18.24	16.71–19.88
3	2,275	120	5.27	4.43–6.27
4	2,275	29	1.27	0.89–1.82
5	2,275	7	0.31	0.15–0.63
6	2,275	2	0.09	0.02–0.32
7	2,275	2	0.09	0.02–0.32
9	2,275	1	0.04	0.01–0.25

^a^Total number of HPV-positive cases.

^b^Number of HPV-positive cases with coinfection by several HPV types.

**Table 2:** Highly prevalent HPV coinfection combinations among females attending a hospital in Zhangzhou.

The most prevalent multiple HPV infection	*N* ^a^	*n* ^b^	%
HPV52 + HPV53	2,275	12	0.52
HPV52 + HPV58	2,275	11	0.48
HPV42 + HPV52	2,275	9	0.39
HPV53 + HPV81	2,275	9	0.39
HPV42 + HPV81	2,275	8	0.35
HPV51 + HPV52	2,275	8	0.35
HPV42 + HPV51	2,275	7	0.31
HPV44 + HPV52	2,275	7	0.31
HPV51 + HPV66	2,275	7	0.31
HPV52 + HPV59	2,275	7	0.31

^a^Total number of HPV-positive cases.

^b^Number of HPV-positive cases with several HPV coinfection patterns.

### 3.2 Genotype-specific prevalence of HPV infection

Among all HPV-positive cases, 1,698 cases were detected as HR-HPV infection (1,698/13,492, 12.58, 95% CI: 12.04–13.16%); 884 cases were detected as LR−/uncertain oncogenic significance HPV infection (884/13,492, 95% CI: 6.15–6.98%). As summarized in Table 3, the genotype-specific HPV prevalence among all participants or among HPV-positive females was calculated.

**Table 3:** Genotype-specific prevalence of HPV infection among all participants and HPV-positive cases.

HPV genotype	No. of HPV-positive cases	Prevalence among all participants (N1 = 13,492)^a^		Prevalence among HPV-positive females (N2 = 2,275)^b^	
	No.	%	95% CI	%	95% CI
HPV16	119	0.88	0.74–1.05	5.23	4.39–6.22
HPV18	72	0.53	0.42–0.67	3.16	2.52–3.97
HPV26	4	0.03	0.01–0.08	0.18	0.07–0.45
HPV31	28	0.21	0.14–0.30	1.23	0.85–1.77
HPV33	53	0.39	0.30–0.51	2.33	1.79–3.03
HPV35	40	0.30	0.22–0.40	1.76	1.29–2.39
HPV39	176	1.31	1.13–1.51	7.74	6.71–8.91
HPV45	26	0.19	0.13–0.28	1.14	0.77–1.66
HPV51	228	1.69	1.49–1.92	10.02	8.86–11.30
HPV52	483	3.58	3.28–3.91	21.23	19.60–22.96
HPV53	237	1.76	1.55–1.99	10.42	9.24–11.73
HPV56	83	0.62	0.50–0.76	3.65	2.96–4.49
HPV58	208	1.54	1.35–1.76	9.14	8.04–10.37
HPV59	92	0.68	0.56–0.84	4.04	3.31–4.92
HPV66	90	0.67	0.54–0.82	3.96	3.24–4.83
HPV68	126	0.93	0.78–1.11	5.54	4.66–6.57
HPV82	26	0.19	0.13–0.28	1.14	0.77–1.66
HPV6	58	0.43	0.33–0.56	2.55	1.97–3.29
HPV11	19	0.14	0.09–0.22	0.84	0.54–1.31
HPV40	47	0.35	0.26–0.46	2.07	1.56–2.73
HPV42	185	1.37	1.19–1.58	8.13	7.07–9.33
HPV43	80	0.59	0.48–0.74	3.52	2.84–4.35
HPV44	121	0.90	0.75–1.07	5.32	4.46–6.33
HPV61	193	1.43	1.24–1.65	8.48	7.40–9.70
HPV81	231	1.71	1.51–1.95	10.15	8.98–11.45
HPV55	46	0.34	0.26–0.45	2.02	1.52–2.67
HPV83	3	0.02	0.01–0.07	0.13	0.04–0.40

^a^These genotype-specific HPV prevalences were calculated using total participants as denominator.

^b^These genotype-specific HPV prevalences were calculated using HPV-positive participants as denominator.

Among 17 kinds of HR-HPV genotype, HPV-52 (3.58%, 483/13,492; 21.23%, 483/2,275) was the most prevalent HPV type, followed by HPV53 (1.76%, 237/13,492; 10.42%, 237/2275), HPV51 (1.69%, 228/13,492; 10.02%, 228/2,275) (Table 3). Among 10 kinds of LR-HPV genotypes, HPV81 exhibited the highest prevalence (1.71%, 231/13,492; 10.15%, 231/2,275), followed by HPV61 (1.43%, 193/13,492; 8.48%, 193/2,275) and HPV42 (1.37%, 185/13,492; 8.13%, 185/2,275) (Table 3). In particular, the sum of 27 genotype-specific HPV prevalence rates (22.78%) in Table 3 exceeded the overall HPV prevalence (16.86%) because some individuals had co-infections with multiple HPV types. Similarly, the sum of the genotype-specific prevalence rates among HPV-positive females in Table 3 exceeded 100% owing to multiple HPV co-infections.

### 3.3 Age-specific HPV prevalence across different age groups

Then, we investigated the association between HPV prevalence and age. As shown in Figure 2A, “two-peaks” age-related trend was observed in the overall HPV prevalence. The first peak in overall HPV prevalence was observed in the ≤29-year age group (19.69%, 103/523). Thereafter, the prevalence gradually declined to the 40–49-year age group (13.64%, 522/3,827). A second peak in the overall prevalence was observed in ≥60-year age group (24.45%, 370/1,513) (Table 4, Figure 2A). To compare the OR across different age groups, we used the prevalence of ≥60-year age group as the reference group. As summarized in Table 5, the overall prevalence in 30-39-year age group (OR = 0.54, 95% CI: 0.46–0.63), 40–49-year age group (OR = 0.49, 95% CI: 0.42–0.57), and 50–59-year age group (OR = 0.68, 95% CI: 0.59–0.78) were significantly lower than the prevalence of ≥60-year age group (*p* < 0.0001). The prevalence in ≤29-year age group (OR = 0.76, 95% CI: 0.59–0.97, *p* = 0.0263) showed no significant difference compared with ≥60-year age group (*p* > 0.005).

**Figure 2:**
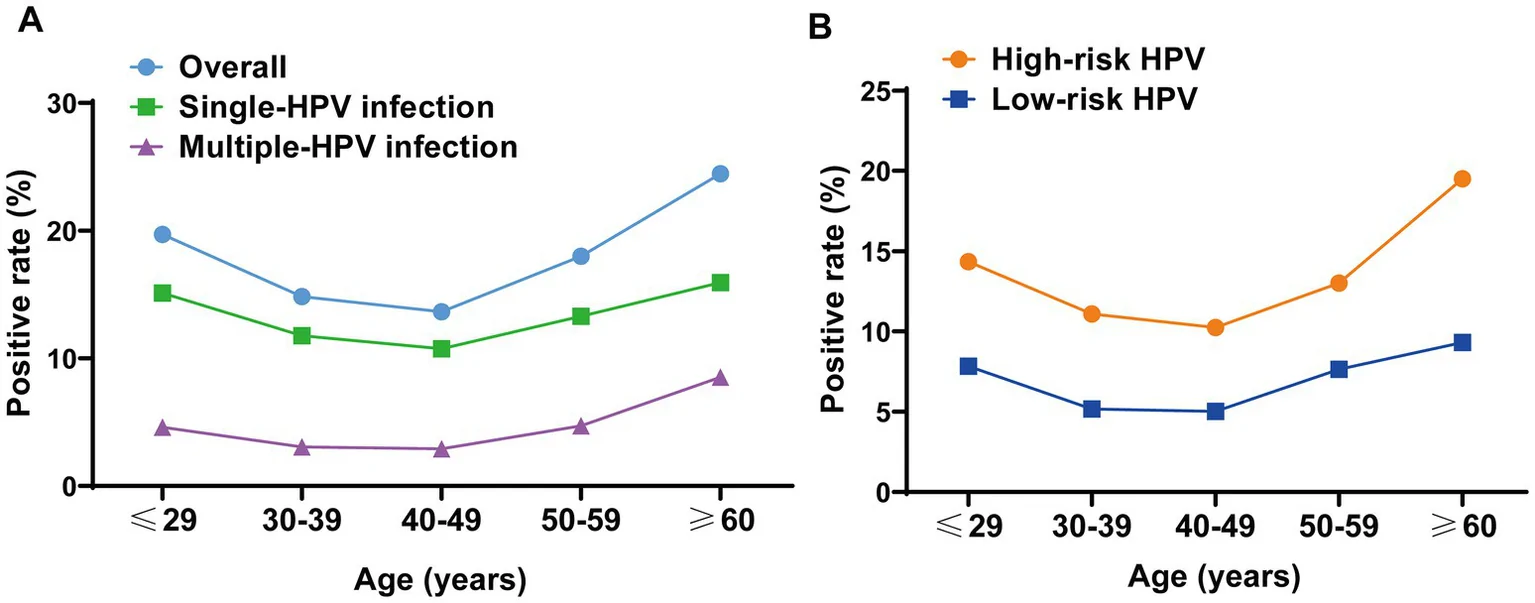
Distribution of HPV prevalence across different age groups: **(A)** Distribution of overall HPV prevalence (blue), single-HPV genotype infection (green), and multiple-HPV infection prevalence (purple) across different age groups. **(B)** Distribution of high-risk HPV prevalence (orange) and low-risk/uncertain oncogenic significance HPV (blue) prevalence across different age groups.

**Table 4:** Age-specific prevalence of overall, single/multiple, and high-risk/low-risk HPV infection among 13,492 females in Zhangzhou.

Age group	*N*	Overall HPV infection (%)	Single infection (%)	Multiple infection (%)	HR-HPV infection (%)	LR-HPV infection (%)
≤29	523	103 (19.69)	79 (15.11)	24 (4.59)	75 (14.34)	41 (7.84)
30–39	2,939	436 (14.84)	346 (11.77)	90 (3.06)	326 (11.09)	152 (5.17)
40–49	3,827	522 (13.64)	410 (10.71)	112 (2.93)	392 (10.24)	192 (5.02)
50–59	4,690	844 (18.00)	622 (13.26)	222 (4.73)	610 (13.01)	358 (7.63)
≥60	1,513	370 (24.45)	242 (15.99)	128 (8.46)	295 (19.50)	141 (9.32)
Total	13,492	2.275 (16.86)	1,699 (12.59)	576 (4.27)	1,698 (12.58)	884 (6.55)
Regression coefficient of age^2^ (95%CI) ^a^		0.00116 (0.0008–0.0015)	0.00068 (0.0003–0.0010)	0.00163 (0.0011–0.0022)	0.00133 (0.0010–0.0017)	0.00078 (0.0003–0.0013)
*p* ^b^		<0.0001	0.0004	<0.0001	<0.0001	0.0014

^a^Regression coefficient and 95% CI of the age^2^ terms in logistic regression models, including age and age^2^ as continuous variables.

^b^*p*-value of age^2^ regression coefficient was calculated to test the statistical significance of non-linear association.

**Table 5:** Odd ratios (ORs) with 95% CI of age-specific HPV prevalence.

Age group	Overall HPV ORs	*p^a^*	Single HPV ORs	*p*	Multiple HPV ORs	*P*	HR-HPV ORs	*p*	LR-HPV ORs	*p*
≤29	0.76 (0.59–0.97)	0.0263	0.93 (0.71–1.23)	0.68	0.52 (0.33–0.80)	0.0036	0.69 (0.53–0.90)	0.0085	0.83 (0.58–1.19)	0.32
30–39	0.54 (0.46–0.63)	<0.0001	0.70 (0.59–0.84)	0.0001	0.34 (0.26–0.45)	<0.0001	0.52 (0.43–0.61)	<0.0001	0.53 (0.42–0.67)	<0.0001
40–49	0.49 (0.42–0.57)	<0.0001	0.63 (0.53–0.75)	<0.0001	0.33 (0.25–0.42)	<0.0001	0.47 (0.40–0.56)	<0.0001	0.51 (0.41–0.64)	<0.0001
50–59	0.68 (0.59–0.78)	<0.0001	0.80 (0.68–0.94)	0.0081	0.54 (0.43–0.67)	<0.0001	0.62 (0.53–0.72)	<0.0001	0.80 (0.66–0.99)	0.04
≥60	Ref		Ref		Ref		Ref		Ref	

^a^*p*-value was calculated using Chi-square or Fisher’s exact test, and test level of *p*-value was adjusted using the Bonferroni method.

We then performed binary logistic regression model, with age and an age-squared term as independent variables and HPV infection status as the dependent variable, to explore the nonlinear relationship between age and overall HPV prevalence. As shown in Figure 3A, a statistically significant nonlinear relationship was observed (quadratic-term regression coefficient = 0.00116, quadratic term *p* < 0.0001) (Table 4) between HPV prevalence and age.

**Figure 3:**
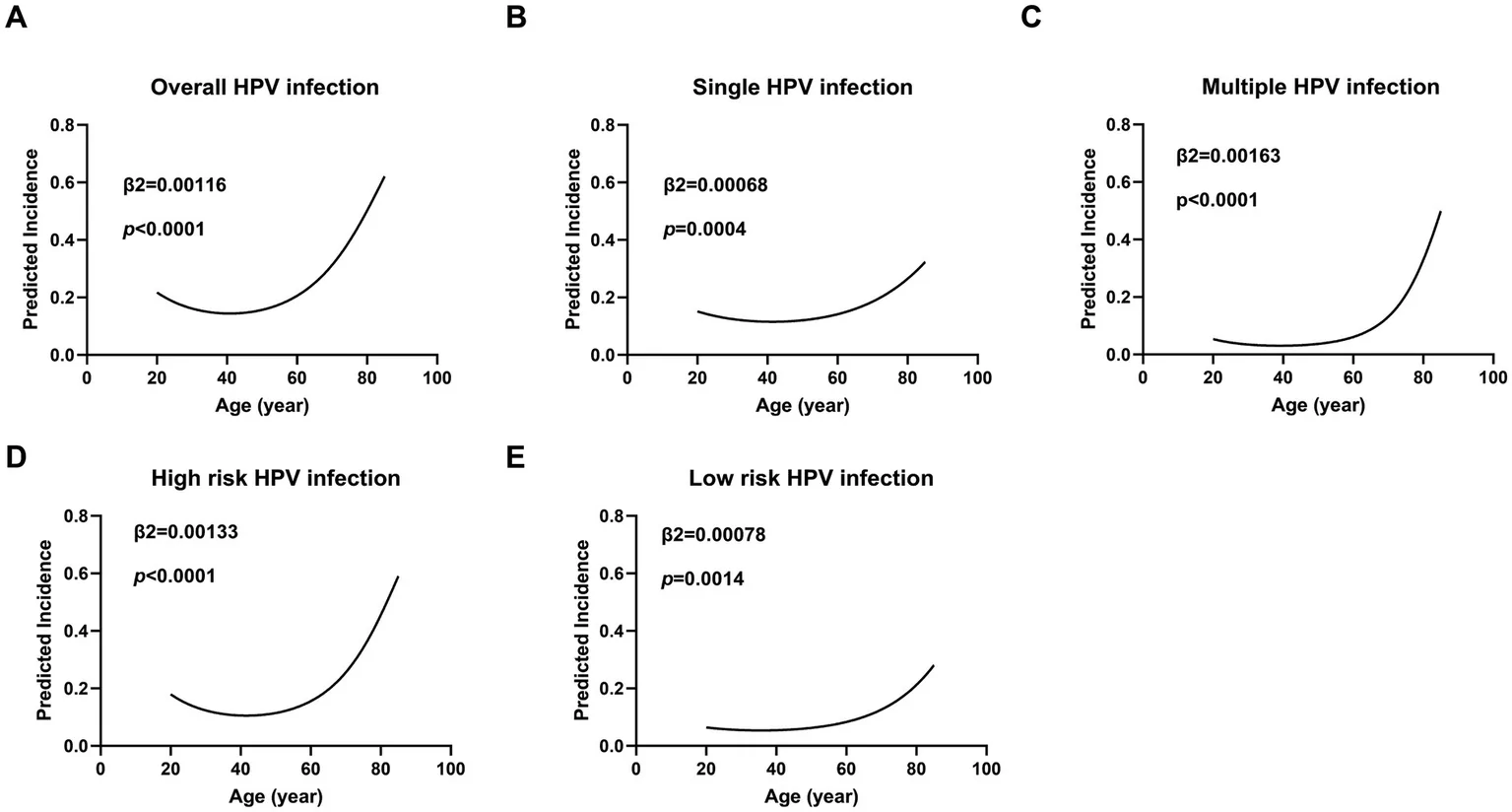
Correlation between HPV prevalence and age analyzed using binary logistic regression. We included *age* and age squared (*age*^2^) terms to analyze their correlation with HPV infection status (positive or negative) using logistic regression. Independent variable: *age* and *age*^2^; dependent variable: HPV infection status. **(A)** The correlation between overall HPV prevalence and *age*. The quadratic term regression coefficient (*β*_2_) = 0.00116, *p* < 0.0001. **(B)** The correlation between single-type HPV prevalence and *age*. The regression coefficient of the quadratic term (*β*_2_) = 0.00068, *p* = 0.0004. **(C)** The correlation between multiple-type HPV prevalence and *age*. The regression coefficient of the quadratic term (*β*_2_) = 0.00163, *p* < 0.0001. **(D)** The correlation between high-risk HPV prevalence and *age*. The regression coefficient of the quadratic term (*β*_2_) = 0.00133, *p* < 0.0001. **(E)** The correlation between low-risk/uncertain oncogenic significance HPV prevalence and *age*. The regression coefficient of the quadratic term (*β*_2_) = 0.00078, *p* = 0.0014; *p* < 0.05 was considered statistically significant.

As shown in Figure 2A, the prevalence of single and multiple HPV infection showed similar age-related trends as overall HPV prevalence, with peak prevalence in the ≥60-year age group and a second peak prevalence in the ≤29-year age group (Table 4). The ORs with 95% CI and the Fisher’s exact test *p*-value of single/multiple HPV infection are summarized in Table 5. Binary logistic regression analysis revealed that the prevalence of single (quadratic-term regression coefficient = 0.00068, quadratic-term *p* = 0.0004) (Figure 3B) and multiple (quadratic-term regression coefficient = 0.00163, quadratic-term *p* < 0.0001) (Figure 3C) HPV infection showed non-linear relationship trends with age (Table 4).

Prevalence of HR-HPV infection and LR-HPV infection also showed similar age-related trends as the prevalence of overall HPV infection (Figure 2B). The first and second peak prevalences of HR-HPV and LR-HPV were also observed in the ≤29-year age group and the ≥60-year age group. The ORs with 95% CI and the Fisher’s exact test *p*-value are summarized in Table 5. Binary logistic regression indicated a nonlinear relationship between HR-HPV prevalence and age (quadratic-term regression coefficient = 0.00133, quadratic term *p* < 0.0001) (Figure 3D). In addition, a non-linear trend with age was also found in the prevalence of LR-HPV (quadratic-term regression coefficient = 0.00078, quadratic term *p* = 0.0014) (Figure 3E; Table 4).

## 4 Discussion

In this study, we investigated the age- and genotype-specific HPV prevalence among females attending the physical examination center at a hospital in Zhangzhou City, Fujian Province, China. Our research identified the predominant HPV types in Zhangzhou and the age-related distribution of HPV prevalence among all participants. Our research provides baseline HPV prevalence data in Zhangzhou, which contributes to the formulation of HPV preventive measures and emphasizes the importance of HPV surveillance, especially for the age groups with high HPV prevalence.

Our data revealed that the overall HPV prevalence in Zhangzhou is 16.86%, which was similar to the overall HPV prevalence in Beijing (17.28%) (8) and the nation-wide research among Chinese women (17.70%) (6). The overall prevalence in our study was significantly lower than that reported in Fujian Province in 2017 (38.3%) (7) and higher than that reported in Zhejiang Province (15.29%) (9). These significant discrepancies may partly reflect the increasing HPV vaccination coverage and the improvement of social HPV-preventive awareness in Fujian Province in recent years. Other potential explanations for the discrepancies should also be considered, including discrepancies in study populations, sampling strategies, age-group distribution, laboratory detection assays, study time windows, cervical screening-seeking behaviors, and additional epidemiological determinants. However, vaccination status and other epidemiological factors were not available for individual participants in this study. Therefore, this hypothesis needs further investigation in the future. These observations suggest that HPV infection remains a public health challenge in some regions of China. In addition, regional disparities in HPV prevalence across China highlight the importance of effective public health measures for the prevention of HPV infection.

We observed that the predominant HR-HPV types in Zhangzhou City were HPV52, HPV53, HPV51, HPV58, and HPV39, respectively. A previous study in Fujian Province reported that HPV16 (8.5%), HPV52 (7.9%), HPV58 (6.2%), and HPV53 (3.5%) accounted for over 60% of all HPV-positive cases (7). Another study in Zhejiang Province demonstrated that HPV-16, HPV-52, HPV-58, and HPV-18 were the predominant HPV types (9). In addition, HPV-52, HPV-16, and HPV-58 were predominant types in Guangzhou (10). Based on these studies, we found that the prevalence of HPV16 and HPV18 was comparatively low in Zhangzhou, which was inconsistent with the findings from other geographical regions of China. Therefore, these results indicate that HPV genotype distribution differs significantly across geographical regions, which may be attributable to regional differences in economic conditions or HPV vaccination programs.

In China, the licensed 9-valent HPV vaccines provide predominant protection against the most prevalent HR-HPV genotypes, including HPV16, 18, 6, 11, 31, 33, 45, 52, and 58 (11, 12). In this study, we observed that the most prevalent HPV type, HPV-52, has been included in the licensed vaccine in China. However, other prevalent HR-HPV types, including HPV53 and HPV51, were not included in the licensed vaccines in China. In addition, the included types HPV16, 18, 31, 33, 45, 6, and 11 exhibited relatively low prevalence in Zhangzhou. These findings reflect only the genotype diversity of local individual HPV infection characteristics and do not indicate the inadequacy or ineffectiveness of the currently available HPV vaccine in China. Therefore, additional local HPV vaccination data need to be collected to further clarify the protective effectiveness of current HPV vaccines against HPV infection in Zhangzhou. In conclusion, these findings underscore the need to consider the relationship between the currently available HPV vaccine and the currently prevalent HPV types. Furthermore, it provides valuable insights for the development of next-generation HPV vaccines.

In our study, we observed that the ≥60-year age group exhibited the highest HPV prevalence, implying that HPV infection still posed a substantial disease burden among older adult females. The females in the ≤29-year age group also showed an elevated HPV prevalence. Therefore, the HPV prevalence among females in Zhangzhou City seemed to exhibit a “two-peak” age-related distribution. Furthermore, logistic regression analysis also revealed a non-linear association between age and HPV infection. These observations were similar to previous research in Zhejiang Province and nation-wide research in China (6, 9). However, the correlation between age and infection rate was only limited to speculation, as a few factors, such as sexual activity (13), changing sexual partners, immune weakening, and vaccine coverage, may influence HPV infection (14–17), have not been taken into account in our research. In conclusion, our data underscore the need for regular HPV screening and surveillance among young and older adult females in Zhangzhou.

Our research still has the following limitations: (1) We only provided basic and limited information about the study population, including age and testing results. Other important population characteristics, including marital status, residence/urban–rural status, previous cervical cancer screening, HPV vaccination status, and history of cervical lesions, may significantly influence HPV infection. Therefore, more detailed information about the study population needs to be collected. (2) The participants in this study are hospital-based, which may differ substantially from the general population. Therefore, our findings may not be applicable to the general population. (3) Our research is a single-center retrospective study. Therefore, these findings may not be generalized to a wider population in Zhangzhou. (4) Cytological and histopathological findings related to cervical lesions and clinical follow-up data were unavailable. Consequently, we could not identify CIN1/CIN2/CIN3 or invasive cervical cancer cases, and could not establish associations between specific HPV genotypes and cervical cancer based on the current data. The conclusions are limited to HPV prevalence and genotype distribution.

In conclusion, this research investigated the prevalence and genotype distribution of HPV infection in females attending the physical examination center of a hospital in Zhangzhou City, China. A non-linear distribution was observed between HPV prevalence and age, with older adult females (≥60 years old) exhibiting the highest HPV infection rate. In addition, HPV52, 53, and 51 were the highly prevalent HR-HPV types in Zhangzhou City. Our results implied that HR-HPV infection still poses a substantial disease burden among older adult females in Zhangzhou City and revealed the prevalent HR-HPV types in Zhangzhou City. This study emphasized the importance of HPV infection surveillance, especially among older adult females, and provided valuable insights into the preventive measures targeting predominant HR-HPV types in Zhangzhou City.

## Data Availability

The original contributions presented in the study are included in the article/supplementary material, further inquiries can be directed to the corresponding author.
